# Use of mobile and cordless phones and cognition in Australian primary school children: a prospective cohort study

**DOI:** 10.1186/s12940-016-0116-1

**Published:** 2016-02-19

**Authors:** Mary Redmayne, Catherine L. Smith, Geza Benke, Rodney J. Croft, Anna Dalecki, Christina Dimitriadis, Jordy Kaufman, Skye Macleod, Malcolm R. Sim, Rory Wolfe, Michael J. Abramson

**Affiliations:** Population Health Research on Electromagnetic Energy (PRESEE), School of Public Health and Preventive Medicine, Monash University, The Alfred Centre, 99 Commercial Road, Melbourne, 3004 Australia; School of Psychology, University of Wollongong, Northfields Avenue, Wollongong, NSW 2522 Australia; Swinburne University of Technology, John Street, Hawthorn, VIC 3122 Australia

**Keywords:** Cognition, Reactions, Accuracy, Mobile phone, Cordless phone, Episodic memory, Spatial ability, Executive ability

## Abstract

**Background:**

Use of mobile (MP) and cordless phones (CP) is common among young children, but whether the resulting radiofrequency exposure affects development of cognitive skills is not known. Small changes have been found in older children. This study focused on children’s exposures to MP and CP and cognitive development. The hypothesis was that children who used these phones would display differences in cognitive function compared to those who did not.

**Methods:**

We recruited 619 fourth-grade students (8-11 years) from 37 schools around Melbourne and Wollongong, Australia. Participants completed a short questionnaire, a computerised cognitive test battery, and the Stroop colour-word test. Parents completed exposure questionnaires on their child’s behalf. Analysis used multiple linear regression. The principal exposure-metrics were the total number of reported MP and CP calls weekly categorised into no use ('None'); use less than or equal to the median amount (‘Some’); and use more than the median (‘More’). The median number of calls/week was 2.5 for MP and 2.0 for CP.

**Results:**

MP and CP use for calls was low; and only 5 of 78 comparisons of phone use with cognitive measures were statistically significant. The reaction time to the response-inhibition task was slower in those who used an MP ‘More’ compared to the ‘Some’ use group and non-users. For CP use, the response time to the Stroop interference task was slower in the ‘More’ group versus the ‘Some’ group, and accuracy was worse in visual recognition and episodic memory tasks and the identification task. In an additional exploratory analysis, there was some evidence of a gender effect on mean reaction times. The highest users for both phone types were girls.

**Conclusions:**

Overall, there was little evidence cognitive function was associated with CP and MP use in this age group. Although there was some evidence that effects of MP and CP use on cognition may differ by gender, this needs further exploration. CP results may be more reliable as parents estimated children’s phone use and the CPs were at home; results for CP use were broadly consistent with our earlier study of older children.

**Electronic supplementary material:**

The online version of this article (doi:10.1186/s12940-016-0116-1) contains supplementary material, which is available to authorized users.

## Background

There is ongoing concern about possible health and/or developmental effects of children’s exposure to radiofrequency electromagnetic fields (RF-EMF) [1, 2]. Children are typically more vulnerable to environmental agents than adults. There have been few studies investigating cognitive effects from school-aged children’s MP use, [3–7], and only one of these included children under 10 years old [6].

We have previously examined 317 older children’s (median age 13) cognition in relation to wireless phone use in the MoRPhEUS study. We found that at baseline more frequent users of MPs displayed shorter reaction times for simple and associative learning tasks but less accurate working memory [5]. The follow-up longitudinal data collected a year later again found shorter reaction times, but less so for those who used a MP more at baseline, and greater for those who had originally had little use, but whose use had increased in the intervening year [7].

Three short-exposure provocation studies with young adolescents had either similar or no effects compared to MoRPhEUS. The first, with three groups aged 13-15, 19-40 and 55-70 years, reported subtle decrements in memory accuracy in the adolescents (but not other age groups) tested during 1-hour ‘3rd Generation’ (3G) exposure, but not with ‘2nd Generation’ (2G) exposure [8]. Reaction times were not affected. The second was a Finnish study of 10-14 year old children (*n* = 32) which found no differences in simple reaction time, or the speed or accuracy of working memory [4]. Cognitive tests were undertaken during exposure/non-exposure to an ‘active’ or ‘inactive’ 902 MHz 2G phone over 50 to 60 min. The third study found 10-12 year olds’ (*n* = 18) simple reaction time was reduced during approximately 30 min’ exposure [3].

Identifying cognitive changes related to MP or CP use, and whether they are likely to be beneficial or detrimental to cognitive development, is particularly important since the age of regular use of RF-EMF emitting devices is decreasing, while the extent of use is increasing, especially among young children [9, 10]. The aims of this ExPOSURE (Examination of Psychological Outcomes in Students using Radiofrequency dEvices) study were to assess current use of mobile and cordless phones in a representative sample of year 4 primary school children (average age 10 years), and to determine whether there was any association between MP or CP use and cognitive function in this age group.

## Methods

### Study design

ExPOSURE was a prospective cohort study, with this paper presenting the baseline cross-sectional results.

In mid-2011, principals of primary schools (independent, state, and Catholic) in the Melbourne metropolitan area and Wollongong were approached with a view to recruiting 600 children. This number was calculated as necessary to give the study sufficient power to detect effects of similar magnitude to those observed in MoRPhEUS, our previous study of cognitive effects in secondary school children [5]. A representative proportion of State, Catholic and private schools were selected by using a computer generated randomisation list from each category. Catholic students were slightly under-represented, but we do not consider this would have introduced any systematic bias. We enrolled 619 students from year 4 classes at 37 primary schools in Melbourne and Wollongong.

Parents were asked to complete a questionnaire, based on that of a 13-country study (the Interphone Study), in relation to their children’s mobile and cordless phone use [11], the extent of use [10], and their child’s health [5]. Children completed a shorter questionnaire reporting only whether they owned or used a MP (but not the extent of use), exercise, and their use of other technology (not reported here). Participating students’ cognitive abilities were measured with the cooperation of schools. All student testing and questionnaires were conducted by a trained member of the research team. Parents filled in their questionnaires at home.

The study was approved by Monash University and University of Wollongong Human Research Ethics Committees, Victorian Department of Education and Early Development, NSW Department of Education and Communities, and Catholic Education Offices. Written informed consent was obtained from participating principals, teachers, parents and students.

### Cognitive tests

Cognitive function was assessed using a computerized psychometric test battery (CogState Research™, Melbourne, 2005, www.cogstate.com) and the Stroop colour/word test [12]. The battery included several well-validated instruments [13] that tested the following cognitive function domains. Those tests used in the Morpheus study that were applicable to this age group were selected to enable direct comparison of results.

1. The Detection (DET) task evaluated simple reaction time and psychomotor speed.
2. The Identification (IDN) task evaluated choice reaction time and assessed visual attention.
3. The One Back task (ONB) evaluated working memory.
4. The One Card Learning (OCL) task assessed visual recognition episodic memory (simple learning).
5. The Go-No Go task (GNG) was a response inhibition task.
6. The Groton Maze Learning Test (GMLT) was a hidden pathway maze learning task assessing spatial and executive ability.

Details of testing administration are available online with additional material. The battery typically took 30 min to complete, which was considered to be within the attention span of 10 year old children. All tasks required participants to respond to stimuli on a computer screen as quickly and accurately as possible. Each test was preceded by a practice run.

The Stroop colour word test involved reading words representing the names of colours [14]. There were four subtasks, the first asking participants to read 50 words written in black ink (Task A). Task C involved identifying colours of meaningless symbols. Tasks B and D asked participants to read out 50 words where the word and colour were incongruous (eg. 'Red' printed in yellow ink) (Task B), and to identify the colour in which the words were printed, ignoring the text itself (Task D). Completion times and error rates were recorded. Others have used the Stroop test in 7 to 9 year old children [15].

Students were eligible to participate if they were attending a year 4 class selected by a participating school, and the student and parent/guardian were able to understand, and were willing to comply with, the information in the plain language sheet and consent form. Thirteen participants (8 male, 5 female) reported to have ADHD were included. The regression analysis was repeated after excluding these children and no differences were seen in the results (not shown). Therefore the ADHD children were retained in the final analysis.

### Statistical analysis

Exposure metrics were the total number of reported voice calls made and received on a MP weekly, the total number of SMS texts sent and received weekly, and the total number of reported voice calls made and received on a CP weekly. Questions asked were of the form: “What is the average number of calls your child makes per week….”, with separate sections for questions on MP and CP use. Some parents gave a range of calls or SMS texts per week, in which case we took the arithmetic mean of that range. The geometric mean may be more suitable when some given ranges are large and start with a small number [16], however no such ranges were given here.

Descriptive raw data were explored, including cognitive results for phone users and non-users. The numbers of reported calls on both kinds of wireless phones were low (see results), so the exposure metrics were split into three groups prior to analysis: no use (None); use but less than or equal to the median amount (Some); and use more than the median (More). SMS use was also very low, so we present results for MP and CP calls only as RF-EMF exposure to the head from texting would be minimal. For the CogState Research™ tests, the raw data were transformed by CogState to create a set of variables considered optimal for analysis and for the detection of cognitive change [17]. For each individual, on each cognitive test, reaction time (“speed”) has been measured by log_10_ transforming the reaction times for correct responses and taking the mean. Student “accuracy” on most cognitive tests was measured using the arcsine transformation of the proportion of correct responses. Only one CogState outcome, accuracy for the Groton Maze Learning domain, was simply the total number of errors made [17] and this variable was subsequently log_e_ transformed for analysis.

Multiple linear regression was used to examine the association of each of the exposure metrics in turn with each of the CogState Research™ outcomes and the Stroop colour/word test derived ratios. Stroop time ratios were derived and analyzed by comparing form B with form A, and form D with form C [18]. Robust standard errors were used to allow for clustering of children by school [19]. As cognitive changes are to be expected during the course of development, we adjusted for age. Previous research has shown differences by gender during the course of normal development in the age-range encompassed here [20, 21]. Interactions between exposure metrics and gender were therefore examined to test for any differences by gender.

Adjustments were also made for the effects of languages other than English, handedness and socioeconomic status. The SocioEconomic Index for Areas (SEIFA), based on post codes, was used as a proxy for socioeconomic status [22]. No adjustments were made for multiple comparisons, but we examined the results for consistent patterns of findings rather than solely focussing on isolated significant *p*-values.

The regression models yielded coefficients (with 95 % confidence intervals) that represented the difference in adjusted outcome means between two exposure groups. For example, for the “Detection” task, the coefficient for ‘Some’ vs the reference group ‘None’, was -0.010, which indicated a lower adjusted mean in the ‘Some’ group than the ‘None’ group. Direct comparisons of ‘More vs Some’ were obtained using model estimates after fitting the regression models and are presented in the text where relevant. Other regressions results are shown in tabular form.

## Results

### Descriptive data

There were 290 (47 %) boys and 329 (53 %) girls, with a mean age 9.9 (SD 0.5) years, representing 52 % of 1189 students invited to participate. The age range was 8.6 to 11.4 years, and 594 (96 %) were aged 9 or 10. A fifth of participants (20 %) spoke a language other than English at home. The majority of participants lived in a high socioeconomic area, based on postcodes (Additional file 1: Figure S1, online). Parental responses indicated that 187 (31 %) of 604 students currently owned or used a MP. The median number of weekly MP calls for users was 2.5 (Interquartile range [IQR] 1-5), which for analysis gave: *n* = 417 for the ‘None” group; *n* = 89 for ‘Some’; and *n* = 88 for ‘More’. The median age of starting to use a MP was 8 (IQR 6.5-9.5) years. Of all those who used a MP, 10 % made and/or received ≥10 calls weekly. Most (75 %) never used a hands-free headset or a vehicle-mounted phone for calls. Of those reported to use a MP, 47 % did not use SMS function.

On the other hand, a CP was used by 470 (80 %) of 584 responders. The median number of weekly CP calls for users was 2 (IQR 1-4), which gave: *n* = 114 for the ‘None” group; *n* = 258 for ‘Some’; and *n* = 210 for ‘More’. Of the 470 CP users, 6 % made and/or received ≥10 calls weekly. The highest user groups were dominated by girls for both MP calls (67 %) and CP calls (61 %). Boxplots of reported MP and CP calls and SMS use are available at Additional file 1: Figure S2, online.

Descriptive statistics for the untransformed response times and accuracies of cognitive tests are given in Table 1, and response times and ratios for the Stroop colour-word test are given in Table 2.

**Table 1:** Cognitive outcomes by level of phone use: descriptive statistics for CogState Research™ tasks (untransformed)

		Mobile phone use/ownership exposure group Median (IQR^a^)			Cordless phone use exposure group Median (IQR^a^)		
Cognitive Test (purpose)	Measure	‘None’^e^ *n* = 415	‘Some’^f^ *n* = 88	‘More’^g^ *n* = 86	‘None’^h^ *n* = 114	‘Some’^i^ *n* = 256	‘More’^j^ *n* = 207
Detection (simple reaction time).	Response^b^	347 (306, 420)	351 (297, 411)	367 (321, 412)	344 (309, 414)	346 (304, 403)	359 (307, 420)
	Accuracy^c^	97 (90,100)	97 (90,100)	96 (90,97)	95 (85, 100)	97 (93, 100)	95 (90, 97)
Identification (choice reaction)	Response^b^	596 (524,689)	576 (514,662)	609 (552,705)	580 (526, 670)	593 (521, 671)	602 (534, 694)
	Accuracy^c^	91 (86, 97)	91 (88, 97)	94 (86, 97)	91 (83, 97)	91 (86, 97)	94 (86, 97)
One-back (working memory)	Response^b^	980 (810, 1125)	979 (763, 1161)	970 (826, 1120)	964 (774, 1114)	948 (795, 1113)	987 (818, 1137)
	Accuracy^c^	88 (72, 94)	89 (76, 95)	84 (69, 94)	86 (67, 94)	86 (77, 94)	86 (72, 94)
One card learning (episodic memory)	Response^b^	1071 (880, 1312)	1116 (824, 1358)	1135 (941, 1303)	1040 (876, 1305)	1052 (884, 1290)	1119 (853, 1320)
	Accuracy^c^	58 (48, 65)	57 (49, 65)	58 (47, 65)	56 (48, 64)	59 (51, 66)	58 (46, 65)
Go-NoGo (response inhibition)	Response^b^	641 (552, 736)	603 (552, 701)	670 (598, 754)	642 (565, 742)	636 (544, 726)	639 (569, 724)
	Accuracy^c^	97 (93, 100)	98 (93, 99)	96 (91, 98)	98 (91, 98)	98 (93, 100)	96 (93, 100)
Groton Maze (spatial & executive ability)	Accuracy^d^	70 (54, 88)	69 (58, 78)	73 (56, 93)	71 (58, 88)	69 (54, 84)	70 (56, 88)

^a^Inter Quartile Range; ^b^Response time in milliseconds for true positive and true negatives; ^c^Accurate hit rate (%); ^d^Total errors; ^e^‘None’ = no MP use; ^f^‘Some’ ≤2.5 MP calls per week; ^g^‘More’ >2.5 MP calls per week; ^h^‘None’ = no CP use; ^i^‘Some’ ≤2 CP calls per week; ^j^‘More’ >2 CP calls per week

**Table 2:** Cognitive outcomes by level of phone use: descriptive statistics for STROOP colour word test

		Mobile phone use/ownership exposure group: Median (IQR^a^)			Cordless phone use exposure group: Median (IQRa)		
Form	Parameter	‘None’^e^ *n* = 412	‘Some’^f^ *n* = 87	‘More’^g^ *n* = 88	‘None’^h^ *n* = 112	‘Some’^i^ *n* = 255	‘More’^j^ *n* = 208
A	Time (s)	26 (24, 30)	27 (24, 30)	26 (23, 30)	27 (24, 30)	26 (24, 30)	26 (24, 29)
B	Time (s)	28 (26,33)	30 (27,34)	28 (25, 32)	30 (25, 34)	28 (25. 32)	29 (26, 33)
C	Time (s)	38 (33, 43)	38 (35, 43)	37 (32, 41)	39 (34, 44)	37 (32, 43)	37 (32,42)
D	Time (s)	65 (55, 77)	65 (57, 74)	65 (56, 75)	65 (58, 78)	65 (55, 75)	64 (56, 74)
(B-A)/A^b^	Time ratio	0.10 (0.02, 0.17)	0.10 (0.03, 0.19)	0.08 (0.01, 0.17)	0.09 (0.01, 0.16)	0.09 (0.01, 0.17)	0.11 (0.04, 0.20)
(D-C)/C^b^	Time ratio	0.71 (0.54, 0.92)	0.67 (0.54, 0.98)	0.74 (0.60, 0 95)	0.66 (0.52, 0.96)	0.71 (0.53, 0.95)	0.73 (0.58, 0.92)

^a^Inter Quartile Range

^b^A positive value is how much longer it takes to respond to the incongruous condition compared to the word in black ink or the colour of a meaningless symbol, expressed as a proportion of the time taken to respond to the latter; ^e^‘None’ = no MP use; ^f^‘Some’ ≤2.5 MP calls per week; ^g^‘More’ >2.5 MP calls per week; ^h^‘None’ = no CP use; ^i^‘Some’ ≤2 CP calls per week; ^j^‘More’ >2 CP calls per week

### Association between use of mobile and cordless phones, and cognitive function

There was little evidence of an association between MP call exposure and cognitive outcomes (Tables 3 and 4). Only 5 of 78 comparisons of phone use with cognitive measures undertaken for the main analysis were statistically significant, as follows: the mean reaction time to the response inhibition task (Go/No Go) was found to be significantly longer (that is, slower reactions) in the ‘More’ group compared to the ‘None’ group (Table 3) and similarly, in the ‘More’ group compared to the ‘Some’ group (coefficient = 0.03, *p* = 0.01) (not shown in tables). An additional exploratory analysis showed evidence of effect modification by gender for this outcome (*p* = 0.001 for interaction) (results by gender are in the additional online material). Reaction times for boys were significantly slower in the ‘More’ group compared to both the ‘None’ and ‘Some’ groups (coefficient = 0.06, *p* < 0.001 for both), but no association was found in girls. Similarly, reaction times to the identification task differed by gender (*p* = 0.02 for interaction) with an effect evident in boys but not girls. For boys, the reaction times were significantly slower in the ‘More’ group compared to the ‘None’ (coefficient = 0.04, *p* = 0.002) and ‘Some’ (coefficient = 0.05, *p* = 0.003) groups.

**Table 3:** Mobile phone use cognitive test results for response time

Test	Skill	Parameter	Call group	Regression coefficient^a^	95 % CI for coefficient	*p*
Detection^b^	Simple reaction time and psychomotor speed	Response time	‘None’	0		
			‘Some’	-0.010	(-0.033, 0.014)	0.41
			‘More’	0.005	(-0.019, 0.030)	0.66
Identification^b^	Choice reaction and visual attention	Response time	‘None’	0		
			‘Some’	-0.009	(-0.032, 0.015)	0.47
			‘More’	0.009	(-0.014, 0.032)	0.42
One-back task^b^	Working memory	Response time	‘None’	0		
			‘Some’	0.001	(-0.024, 0.027)	0.93
			‘More’	-0.003	(-0.022, 0.016)	0.77
Go/NoGo^b^	Response inhibition	Response time	‘None’	0		
			‘Some’	-0.001	(-0.020, 0.019)	0.96
			‘More’	**0.029**	**(0.003, 0.054)**	**0.03**
One-card learning^b^	Visual recognition and episodic memory	Response time	‘None’	0		
			‘Some’	0.008	(-0.026, 0.043)	0.63
			‘More’	0.003	(-0.034, 0.039)	0.87
Stroop A^c^		Response time ratio	‘None’	0		
			‘Some’	0.008	(-0.020, 0.036)	0.55
			‘More’	-0.008	(-0.040, 0.024)	0.63
Stroop C^d^		Response time ratio	‘None’	0		
			‘Some’	-0.010	(-0.085, 0.066)	0.79
			‘More’	0.018	(-0.052, 0.087)	0.61

^a^These are regression coefficients adjusted for age, gender, language other than English, handedness, and socioeconomic status. The coefficient represents the difference in adjusted means of the outcome between each of the exposure groups ‘Some’ and ‘More’ and the non-exposed reference group ‘None’. For example, for the simple reaction time for the “Detection” task, the ‘Some’ coefficient is -0.010. This indicates the adjusted mean is lower in the ‘Some’ group than the ‘None’ group

^b^Base 10 log transformed data originally in milliseconds (response time tests)

^c^Time ratio (B-A)/A

^d^Time ratio (D-C)/C

Statistically significant results are in **bold font**

**Table 4:** Mobile phone use cognitive test results for accuracy

Test	Skill	Parameter	Call group	Regression coefficient^a^	95 % CI for coefficient	*p*
Detection^b^	Simple reaction time and psychomotor speed	Accuracy	‘None’			
			‘Some’	0.007	(-0.087, 0.100)	0.89
			‘More’	-0.004	(-0.061, 0.053)	0.89
Identification^b^	Choice reaction and visual attention	Accuracy	‘None’			
			‘Some’	0.018	(-0.036, 0.073)	0.50
			‘More’	-0.012	(-0.077, 0.052)	0.70
One-back task^c^	Working memory	Accuracy	‘None’			
			‘Some’	0.033	(-0.024, 0.091)	0.24
			‘More’	-0.035	(-0.094, 0.024)	0.23
One-card learning^c^	Visual recognition and episodic memory	Accuracy	‘None’			
			‘Some’	0.005	(-0.032, 0.042)	0.78
			‘More’	-0.026	(-0.074, 0.023)	0.30
Groton Maze Learning^d^	Spatial and executive ability	Accuracy	‘None’			
			‘Some’	-0.036	(-0.093, 0.022)	0.21
			‘More’	0.021	(-0.057, 0.100)	0.58
Go/NoGo^c^	Response inhibition	Accuracy	‘None’			
			‘Some’	0.025	(-0.033, 0.083)	0.39
			‘More’	-0.042	(-0.108, 0.025)	0.21

^a^These are regression coefficients adjusted for age, gender, language other than English, handedness, and socioeconomic status. The coefficient represents the difference in adjusted means of the outcome between each of the exposure groups ‘Some’ and ‘More’ and the non-exposed reference group ‘None’. For example, for the accuracy in the “Detection” task, the ‘Some’ coefficient is 0.007and the ‘More’ coefficient -0.004. This indicates the adjusted mean is higher in the ‘Some’ group but lower in ‘More’ group when compared to the ‘None’ group

^b^Base 10 log transformed data originally in milliseconds (response time tests)

^c^Square root arcsine transformed data (accuracy tests)

^d^Base e log transformed (total number of errors)

^e^Time ratio (B-A)/A

^f^Time ratio (D-C)/C

As for MP use, there was little evidence of an association between CP call exposure and cognitive outcomes (Tables 5 and 6). ‘More’ to ‘Some’ comparisons are not shown in the tables, however response time to the Stroop A ((B-A)/A) interference task was slower in the ‘More’ group compared to the ‘Some’ group (coefficient = 0.035, *p* = 0.007), and accuracy was worse in those who made and received ‘More’ CP calls compared to ‘Some’ calls in the OCL task (visual recognition and episodic memory) (coefficient = -0.03, *p* = 0.02) and the identification task (coefficient = -0.06, *p* = 0.05).

**Table 5:** Cordless phone use cognitive test results for response time

Test	Skill	Parameter	Call group	Regression coefficient^a^	95 % CI for coefficient	*p*
Detection^b^	Simple reaction time and psychomotor speed	Response time	‘None’	0		
			‘Some’	-0.010	(-0.043, 0.023)	0.56
			‘More’	0.007	(-0.026, 0.041)	0.66
Identification^b^	Choice reaction and visual attention	Response time	‘None’	0		
			‘Some’	0.003	(-0.014, 0.020)	0.74
			‘More’	0.006	(-0.018, 0.030)	0.60
One-back task^b^	Working memory	Response time	‘None’	0		
			‘Some’	-0.001	(-0.027, 0.025)	0.95
			‘More’	0.006	(-0.025, 0.037)	0.68
Go/NoGo^b^	Response inhibition	Response time	‘None’	0		
			‘Some’	-0.001	(-0.022, 0.020)	0.92
			‘More’	0.007	(-0.019, 0.033)	0.61
One-card learning^b^	Visual recognition and episodic memory	Response time	‘None’	0		
			‘Some’	-0.005	(-0.041, 0.031)	0.79
			‘More’	-0.011	(-0.052, 0.030)	0.59
Stroop A^c^		Response time ratio	‘None’	0		
			‘Some’	-0.006	(-0.041, 0.029)	0.74
			‘More’	0.029	(-0.007, 0.065)	0.11
Stroop C^d^		Response time ratio	‘None’	0		
			‘Some’	0.041	(-0.038, 0.119)	0.30
			‘More’	0.037	(-0.023, 0.097)	0.22

^a^These are regression coefficients adjusted for age, gender, language other than English, handedness, and socioeconomic status. The coefficient represents the difference in adjusted means of the outcome between each of the exposure groups ‘Some’ and ‘More’ and the non-exposed reference group ‘None’. For example, for the simple reaction time for the “Detection” task, the ‘Some’ coefficient is -0.010. This indicates the adjusted mean is lower in the ‘Some’ group than the ‘None’ group

^b^Base 10 log transformed data originally in milliseconds (response time tests)

^c^Time ratio (B-A)/A

^d^Time ratio (D-C)/C

**Table 6:** Cordless phone use cognitive test results for accuracy

Test	Skill	Parameter	Call group	Regression coefficient^a^	95 % CI for coefficient	*p*
Detection^b^	Simple reaction time and psychomotor speed	Accuracy	‘None’	0		
			‘Some’	0.084	(-0.004, 0.172)	0.06
			‘More’	0.012	(-0.057, 0.082)	0.72
Identification^b^	Choice reaction and visual attention	Accuracy	‘None’	0		
			‘Some’	0,032	(-0.020, 0.084)	0.22
			‘More’	-0.024	(-0.075, 0.028)	0.36
One-back task^c^	Working memory	Accuracy	‘None’	0		
			‘Some’	0.023	(-0.049, 0.096)	0.52
			‘More’	-0.022	(-0.095, 0.052)	0.55
One-card learning^c^	Visual recognition and episodic memory	Accuracy	‘None’	0		
			‘Some’	0.009	(-0.024, 0.041)	0.58
			‘More’	-0.022	(-0.058, 0.014)	0.23
Groton Maze Learning^d^	Spatial and executive ability	Accuracy	‘None’	0		
			‘Some’	0.004	(-0.071, 0.079)	0.92
			‘More’	0.054	(-0.031, 0.139)	0.21
Go/NoGo^c^	Response inhibition	Accuracy	‘None’	0		
			‘Some’	0.022	(-0.046, 0.090)	0.52
			‘More’	-0.017	(-0.086, 0.052)	0.62

^a^These are the regression coefficients adjusted for age, gender, language other than English, handedness, and socioeconomic status. The coefficient represents the difference in adjusted means of the outcome between each of the exposure groups ‘Some’ and ‘More’ and the non-exposed reference group ‘None’. For example, for the accuracy in the “Detection” task, the ‘Some’ coefficient is 0.084 and the ‘More’ coefficient 0.012. This indicates the adjusted mean is higher in both the ‘Some’ group and the ‘More’ group when compared to the ‘None’ group

^b^Base 10 log transformed data originally in milliseconds (response time tests)

^c^Square root arcsine transformed data (accuracy tests)

^d^Base e log transformed (total number of errors)

^e^Time ratio (B-A)/A

^f^Time ratio (D-C)/C

The reaction time in the Stroop A ((B-A)/A) interference task differed by gender (*p* = 0.005 for interaction). The reaction time was significantly slower in girls among higher users compared to the ‘None’ (coefficient = 0.07, *p* = 0.002) and ‘Some’ (coefficient = 0.04, *p* = 0.02) groups but no effect was seen in boys.

There were no statistically significant associations between texting and cognition (results not shown). Although gender was only found to be an effect modifier for a few of the cognitive outcomes in this study, gender itself was still consistently a strong predictor of most outcomes, showing strong cognitive differences in this age group (results not reported).

There was no association between parents’ perception of risk and their reported extent of their child’s MP use (*p* = 0.43).

Additional file 1: Figure S3 (online) displays the relationships between phone use and cognitive test measurement for males and females, where evidence of an interaction with gender was found.

### Discrepancy between parent and child exposure measurements

**Fig. 1:**
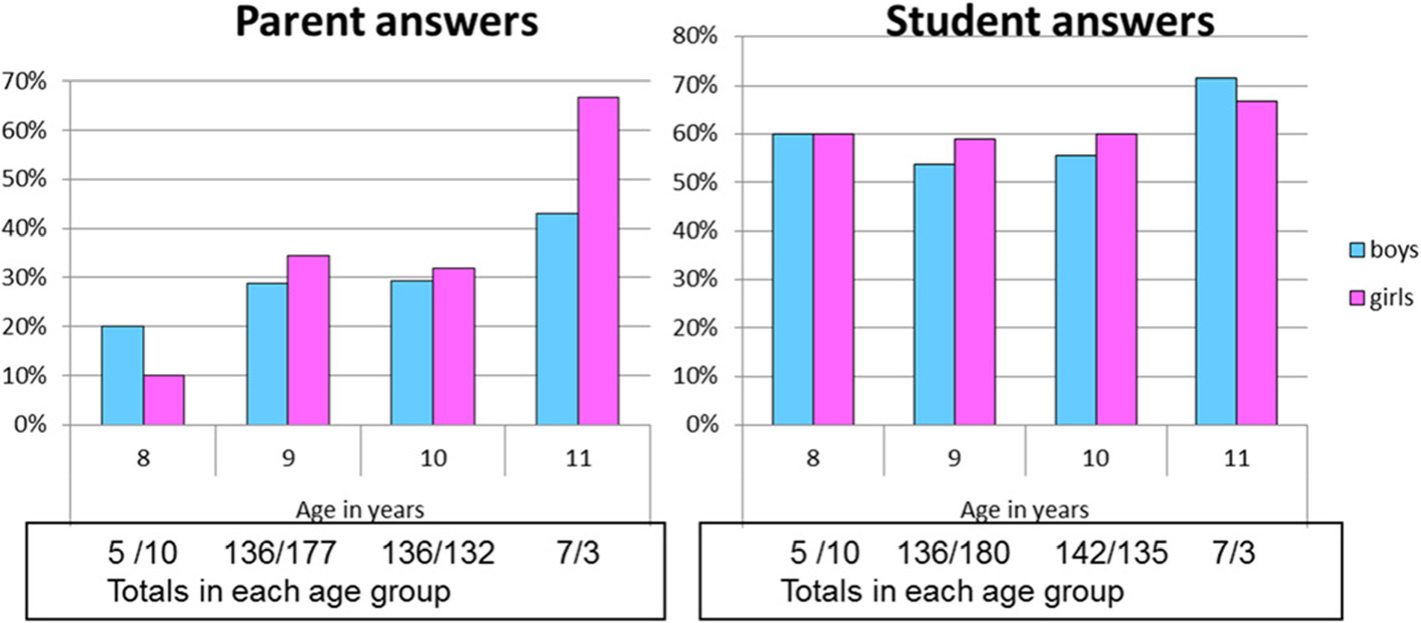
Parent and student responses on student mobile phone ownership: stratified by age. Legend: The proportions of parents and students reporting ownership or use of a mobile phone by students: stratified by age. Many more students reported owning or using a mobile phone than their parents reported suggesting use/exposure of which parents are unaware

Parents were asked “does your child currently own or use a mobile phone”. Children were asked in two questions “do you currently own a mobile phone” and “do you currently use a mobile phone”. For the 603 students where both the child and parent responded, 187 (31 %) of parents indicated their child currently owned or used a MP. This was significantly lower (*p* < 0.001) than the 343 (57 %) of children who said they owned or used one (Fig. 1). This represented agreement for 485 (64 %) respondents and disagreement for 218 (36 %). For the 218 discordant responses, 187 parents said the child did not own or use a phone. Of these, 20 % of children said they owned a MP and 95 % said they used one. A sensitivity analysis for the association between MP calls and cognitive outcomes is given for the discordant and concordant subsamples in Additional file 1: Tables S1 and 2, available online.

## Discussion

There was very little evidence of a consistent association between MP or CP use with cognitive outcomes in this study. This is perhaps not surprising considering the very low level of use by most students. However, there was some evidence that the effect of MP and CP use on cognition may differ between boys and girls in this age group, with reaction times for boys who were higher MP users slower than for those who are low users and non-users for response inhibition and identification tasks; with no association seen in girls. For CP use, reaction time for the Stroop A interference task was significantly slower in girls among higher users compared to both low and non-users, with no association in boys. In our previous study [5] there was some evidence that associations between MP calls and accuracy of working memory were stronger in boys than girls. Given the inconsistencies between the MP and CP results, it is possible that the few significant differences which were observed may have been chance findings. Further the results stratified by gender should be seen as exploratory and hypothesis generating.

CP results may be more accurate as parents estimated children’s phone use and the CP was at home where the parent was more likely to know if it was being used.

Previous studies in adolescents have tended to indicate somewhat faster reactions, but poorer memory, although not consistently with relation to the same cognitive functions (see introduction). The current findings tended in the opposite direction to those with older participants, although the Stroop test had similar results for MP users in the Morpheus study as for CP users in this study. It is not possible to tell from our data whether these differences and similarities are related to different cognitive responses to RF-EMF exposure in younger children than older children, or chance outcomes. Significant gender-specific differences have also been reported in the P600 index using an EEG without RF-EMF exposure, but there were no significant differences with exposure [23]. This index is thought to be related to working memory operations such as rule-governed sequences. Slower reaction times may carry implications for daily activities where a rapid response is necessary for safety reasons. It should be noted that even statistically significant differences were very small.

The literature reveals inconsistencies in gender effects for cognitive tests of memory and reaction time in non-phone use contexts. A few RF-EMF exposure studies have shown gender-dependent differences in electroencephalogram (EEG) spectral power coherence [24], spectral power intensity [25], and amplitude response [23, 26]. However, Papageorgiou et al. found no gender differences in memory performance [26].

In the current study, parents were asked to estimate their child's MP and CP use because a pilot study indicated that many younger children could not recall their use and many were not able to work it out.

It is possible the results suffered from differential misclassification. Since fewer parents knew of MP use by their child than their children reported, it meant we only collected reported use data for approximately half of those students who reported using a MP. Khorseva et al. [6] found the same problem as the children were ‘extensively’ using other people’s phones without their parents’ knowledge. This was overcome in their subsequent, longitudinal, data collection by “cross-questioning” parents with their children, but this would not have been practical for a study of the present size.

We successfully recruited the target sample size of 600 children from two cities, and the study was adequately powered to detect an effect of similar magnitude to that which we observed in the MoRPhEUS study.

It is increasingly difficult in observational research to assess specific RF-EMF exposure effects as artificial environmental exposures from several sources are omnipresent in most settings. At the time of data collection, these included base stations, television and radio antennae and WiFi. However, the majority of a child’s RF-EMF exposure comes from personal devices rather than environmental sources due to the rapid increase as one nears a transmitter. At the time of our data collection, fewer children owned smartphones than currently and fewer schools had ‘Bring Your Own Device’ policies. This study only specifically considered MP and CP exposures, but the increasingly widespread use of other devices for games and at school increases the need to account for these other exposures in future studies. Without this, effects may be diluted, making it harder to identify associations.

Future research considering young children’s use of technology could benefit from new approaches. The amount of personal phone use can most accurately be recorded using a suitable installed phone-based application [27]. Now that phones have multiple functions, it is important for questions to identify the proportion of different types of use e.g. use for calls or social media versus use for games, and their proximity to the body for each use. Sampling the total exposures from all sources in a sample subset is also becoming necessary.

## Conclusions

With only 5 of 78 statistical comparisons being statistically significant, there was little evidence that cognitive function was consistently associated with CP and MP use in this age group. Although exploratory analysis gave some evidence that effects of MP and CP use on cognition may differ between boys and girls, further study is needed to validate this hypothesis.

Of the significant results, those for CP use were more consistent with our earlier study of older children while those for MP use were not. CP results may be more reliable as parents estimated children’s phone use and the CP was at home while the MP was not.

Unexpectedly, many children who were reported by their parent as not using a MP reported themselves that they were doing so. This is an important aspect for future studies to take into account. Ideally, future studies should directly measure exposure.

## Additional file

Additional file 1:
**Further material on cognitive tests, additional results and sensitiv analyses for Exposure study "Use of mobile and cordless phones and cognitive effects in Australian primary school children".** (DOC 104 kb)

## Abbreviations

ADHD: Attention Deficit Hyperactivity Disorder
CP: cordless phone
DET: detection task
Exposure: Examination of Psychological Outcomes in Students using Radiofrequency dEvices
GMLT: Groton Maze learning task
GNG: Go-No Go task
GSM: Global System for Mobile Communications
IDN: Identification task
MHz: Megahertz
MoRPhEUS: Mobile Radiofrequency Phone Exposed Users’ Study
MP: mobile phone
OCL: One Card Learning
ONB: One-back task
RF-EMF: radiofrequency electromagnetic fields
SEIFA: SocioEconomic Index for Areas
