# Supplementary material for: Use of mobile and cordless phones and cognition in Australian primary school children: a prospective cohort study
Source: Environ Health. 2016 Feb 19;15:26. doi: 10.1186/s12940-016-0116-1 (PMC4759913; doi:10.1186/s12940-016-0116-1)
Supplement: Additional file 1: — Further material on cognitive tests, additional results and sensitiv analyses for Exposure study "Use of mobile and cordless phones and cognitive effects in Australian primary school children". (DOC 104 kb) [file 12940_2016_116_MOESM1_ESM.doc]

Additional file for

**Use of mobile and cordless phones and cognitive effects in Australian primary school children**

Mary Redmayne^1^; Catherine L. Smith^1^; Geza Benke^1^; Rodney Croft ^12^; Anna Dalecki^2^; Christina Dimitriadis^1^; Jordy Kaufman^4^; Skye Macleod; Malcolm R. Sim^1^; Rory Wolfe^1^; Michael J. Abramson^1^

**Additional details on the cognitive tests**

*Cognitive Tests*

Cognitive function was assessed using a computerized psychometric test battery (CogState Research™, Melbourne, 2005, www.cogstate.com) and the Stroop colour/word test[^11^](#_ENREF_11). The battery included several well-validated instruments[^12^](#_ENREF_12) that tested the following cognitive function domains.

(1) The Detection (DET) task evaluated simple reaction time and psychomotor speed. A playing card was presented face-down centrally and required the participant to respond YES (with a press of the keyboard K key) as soon as it turned face-up. About 35 trials were recorded and reaction time (speed) was used as the outcome measure as previously described.

(2) The Identification (IDN) task evaluated choice reaction time and assessed visual attention. The participant was required to respond YES if the face-up card was red, or NO (by the keyboard D key) if the card was not red. About 30 trials were recorded, and speed was the outcome measure.

(3) The One Back task (ONB) evaluated working memory. This was similar to the IDN task, except that the participant was required to respond YES if the face-up card was the same as the previous card, and NO if the card was not exactly the same. The outcome measures were speed and accuracy.

(4) The one card learning (OCL) task assessed visual recognition episodic memory. It was similar to the ONB task except the participant was required to respond YES if they had seen the face-up card before in the current test, or NO if they had not. The outcome measures were accuracy and reaction time.

(5) The Go-No Go task (GNG) was a response inhibition task. It was similar to the DET task except that a particular face-up card required a YES response whilst other cards required a withheld response; speed and accuracy were the outcome measures.

(6) The Groton Maze Learning Test (GMLT) was a hidden pathway maze learning task assessing spatial and executive ability. A 10x10 grid of tiles was presented and the participant was required to click on tiles with pre-defined rules in order to find and learn a hidden pathway on 4 trials; total errors was the outcome measure.

**Additional Figure 1** Distribution of socio-economic status, based on home post codes


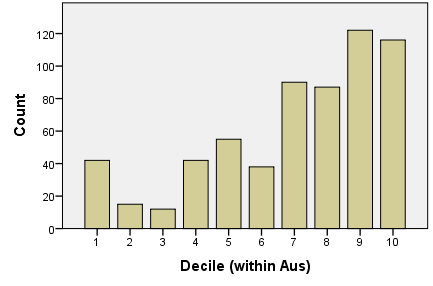


**Additional Figure 2** Boxplots of total reported voice calls per week on wireless phones and total SMS texts sent. The box represents the interquartile range (IQR) and the horizontal line the median value. Outliers (cases with values 1.5-3 times the IQR above the 75^th^ percentile) are represented by ‘∘’ and extreme observation cases with values more than three times the IQR) by ‘*’. Note, 2 extreme outliers are not shown in the MP call graph.


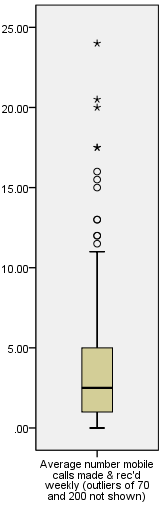

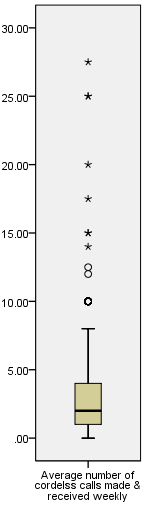

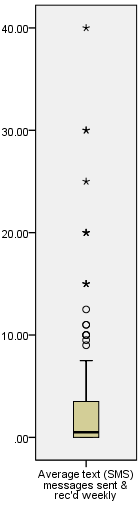


**Additional Figure 3** Estimated relationship between phone use and cognitive test measurement for males and females, where an interaction with gender had been found

For mobile phone use (MP calls), the reaction time in the response inhibition (Top graph: Go/No Go) and identification tasks (central graph) was slower in boys who used MPs than low or no users, but no association was found in girls. For cordless phone use (CP calls), reaction time in the Stroop A ((B-A)/A) interference task was slower in girls that used phones, with no effect evident in boys.

**Additional Table 1. Sensitivity analysis: Association between mobile phone use and cognitive test response times within subsamples defined by concordance or discordance between parents and children’s responses to ownership or use of MP phone**

|  |  |  |  | Concordant responses (n=385) | | | Discordant responses (n=218) | | |
| --- | --- | --- | --- | --- | --- | --- | --- | --- | --- |
| Test | Skill | Parameter | Call group | Regression  coefficient^◊^ | 95% CI for coefficient | p | Regression  coefficient^◊^ | 95% CI for coefficient | p |
| Detection † | Simple reaction time and psychomotor speed | Response time | ‘None’  ‘Some’  ‘More’ | 0  -0.009  0.004 | (-0.038, 0.020)  (-0.020, 0.028) | 0.54  0.73 | 0  -0.006  0.029 | (-0.070, 0.058)  (-0.039, 0.096) | 0.86  0.39 |
| Identification † | Choice reaction and visual attention | Response time | ‘None’  ‘Some’  ‘More’ | 0  -0.009  0.003 | (-0.040, 0.022)  (-0.021, 0.026) | 0.56  0.83 | 0  -0.005  **0.057** | (-0.042, 0.033)  **(0.005, 0.109)** | 0.81  **0.03** |
| One-back task † | Working memory | Response time | ‘None’  ‘Some’  ‘More’ | 0  -0.006  -0.009 | (-0.036, 0.025)  (-0.031, 0.013) | 0.70  0.43 | 0  0.022  0.016 | (-0.023, 0.067)  (-0.050, 0.082) | 0.34  0.63 |
| Go/NoGo † | Response inhibition | Response time | ‘None’  ‘Some’  ‘More’ | 0  0.004  **0.028** | (-0.021, 0.028)  **(0.005, 0.051)** | 0.77  **0.02** | 0  -0.006  0.042 | (-0.051, 0.038)  (-0.031, 0.115) | 0.78  0.25 |
| One-card learning † | Visual recognition and episodic memory | Response time | ‘None’  ‘Some’  ‘More’ | 0  -0.001  -0.008 | (-0.042, 0.041)  (-0.048, 0.032) | 0.98  0.69 | 0  0.025  0.010 | (-0.040, 0.090)  (-0.059, 0.080) | 0.44  0.76 |
| Stroop A * |  | Response time ratio | ‘None’  ‘Some’  ‘More’ | 0  0.023  0.004 | (-0.004, 0.050)  (-0.029 0.038) | 0.09  0.79 | 0  -0.007  -0.012 | (-0.072, 0.058)  (-0.159, 0.134) | 0.83  0.87 |
| Stroop C** |  | Response time ratio | ‘None’  ‘Some’  ‘More’ | 0  0.004  0.035 | (-0.091, 0.098)  (-0.036, 0.107) | 0.94  0.32 | 0  -0.029  -0.088 | (-0.125, 0.066)  (-0.282, 0.105) | 0.54  0.36 |

◊ These are regression coefficients adjusted for age, gender, language other than English, handedness, and socioeconomic status. The coefficient represents the difference in adjusted means of the outcome between each of the exposure groups ‘Some’ and ‘More’ and the non-exposed reference group ‘None’.

† Base 10 log transformed data originally in milliseconds (response time tests)

# Square root arcsine transformed data (accuracy tests)

+ Number of total errors

*Time ratio (B-A)/A

**Time ratio (D-C)/C

Statistically significant results are in **bold font**

**Additional Table 2. Sensitivity analysis: Association between mobile phone use and cognitive test accuracy within subsamples defined by concordance or discordance between parents and children’s responses to ownership or use of MP phone**

|  |  |  |  | Concordant responses (n=385) | | | Discordant responses (n=218) | | |
| --- | --- | --- | --- | --- | --- | --- | --- | --- | --- |
| Test | Skill | Parameter | Call group | Regression  coefficient^◊^ | 95% CI for coefficient | p | Regression  coefficient^◊^ | 95% CI for coefficient | p |
| Detection † | Simple reaction time and psychomotor speed | Accuracy | ‘None’  ‘Some’  ‘More’ | 0  0.019  0.001 | (-0.072, 0.110)  (-0.065, 0.066) | 0.68  0.99 | 0  -0.058  -0.038 | (-0.262, 0.146)  (-0.150, 0.074) | 0.57  0.50 |
| Identification † | Choice reaction and visual attention | Accuracy | ‘None’  ‘Some’  ‘More’ | 0  -0.001  -0.008 | (-0.072, 0.070)  (-0.083, 0.066) | 0.97  0.83 | 0  0.080  -0.016 | (-0.019, 0.179)  (-0.259, 0.227) | 0.11  0.89 |
| One-back task # | Working memory | Accuracy | ‘None’  ‘Some’  ‘More’ | 0  0.004  -0.048 | (-0.052, 0.060)  (-0.119, 0.023) | 0.88  0.18 | 0  0.096  -0.004 | (-0.019, 0.211)  (-0.181, 0.173) | 0.10  0.96 |
| One-card learning # | Visual recognition and episodic memory | Accuracy | ‘None’  ‘Some’  ‘More’ | 0  -0.001  -0.038 | (-0.040, 0.038)  (-0.089, 0.013) | 0.96  0.14 | 0  0.009  0.047 | (-0.061, 0.080)  (-0.060, 0.155) | 0.80  0.38 |
| Groton Maze Learning + | Spatial and executive ability | Accuracy | ‘None’  ‘Some’  ‘More’ | 0  -0.043  0.032 | (-0.105, 0.019)  (-0.061, 0.125) | 0.16  0.49 | 0  0.017  -0.081 | (-0.131, 0.165)  (-0.312, 0.151) | 0.82  0.49 |
| Go/NoGo # | Response inhibition | Accuracy | ‘None’  ‘Some’  ‘More’ | 0  0.002  **-0.075** | (-0.058, 0.061)  **(-0.148, -0.002)** | 0.95  **0.04** | 0  0.029  **0.116** | (-0.117, 0.174)  **(-0.001, 0.233)** | 0.69  **0.05** |

◊ These are regression coefficients adjusted for age, gender, language other than English, handedness, and socioeconomic status. The coefficient represents the difference in adjusted means of the outcome between each of the exposure groups ‘Some’ and ‘More’ and the non-exposed reference group ‘None’.

† Base 10 log transformed data originally in milliseconds (response time tests); # Square root arcsine transformed data (accuracy tests); + Base e log transformed (total number of errors); *Time ratio (B-A)/A; **Time ratio (D-C)/C
